# Source tracing and contagion measurement of carbon emission trading price fluctuation in China from the perspective of major emergencies

**DOI:** 10.1371/journal.pone.0298811

**Published:** 2024-03-08

**Authors:** Binhong Wu, Hongyu Wang, Bangsheng Xie, Zhizhong Xie

**Affiliations:** 1 College of Computer and Information sciences, Fujian Agriculture and Forestry University, Fuzhou, China; 2 College of Economics and Management, Fujian Agriculture and Forestry University, Fuzhou, China; 3 Research Institute of Xi Jinping Ecological Civilization, Fujian Agriculture and Forestry University, Fuzhou, China; 4 Tan Siu Lin Business School, Quanzhou Normal University,Quanzhou,China; University of Galway, Ireland / Anhui University of Finance and Economics, CHINA

## Abstract

Based on monthly economic data spanning from January 2015 to December 2022, we have established an analytical framework to examine the "Russia-Ukraine conflict—financial market pressure and energy market—China carbon emission trading prices." To achieve this objective, we developed indices for financial system pressure, the energy market, and investor sentiment, applying a mediation effects model to validate their transmission mechanisms. Subsequently, the TVP-SV-VAR model was employed to scrutinize the nonlinear impact of the Russia-Ukraine conflict on the valuation of China’s carbon emission trading rights. This model integrates time-varying parameters (TVP) and stochastic volatility (SV), utilizing Markov Chain Monte Carlo (MCMC) technology for parameter estimation. Finally, various wavelet analysis techniques, including continuous wavelet transform, cross-wavelet transform, and wavelet coherence spectrum, were applied to decompose time series data into distinct time-frequency scales, facilitating an analysis of the lead-lag relationships within each time series. The research outcomes provide crucial insights for safeguarding the interests of trading organizations, refining the structure of the carbon market, and mitigating systemic risks on a global scale.

## Introduction

The Russia-Ukraine conflict, recognized as the most significant military confrontation in Europe since the onset of the 21st century, has not only heightened global geopolitical risks and accelerated the transformation of the economic landscape but has also triggered a severe supply crisis and market volatility in commodity trade. Governments now find themselves navigating between resuming conventional fossil energy initiatives, such as coal power, and expanding investments in clean energy to expedite the energy transition. This dynamic presents substantial challenges and uncertainties to the global pursuit of carbon neutrality. Consequently, comprehending the impact mechanism of the Russian-Ukrainian conflict on the carbon emissions trading market becomes crucial for realizing the "double carbon" goal. The fluctuation in carbon emissions trading prices is intricately linked to the progression of the global energy transition toward green and low-carbon practices. Therefore, investigating this impact and formulating corresponding policy responses within a cohesive framework emerges as urgent and vital issues.

Studies has indicated that the Russia-Ukraine conflict involves intricate interactions across geopolitical, international relations, social security, and market economic dimensions [1, 2]. On the one hand, Russia occupies a substantial role in the global energy market, and the conflict has significantly disrupted the production and service supply of specific energy commodities. This disruption has resulted in pronounced fluctuations in global energy prices, and the interdependence between energy prices and carbon emissions trading implies a linkage effect between the two markets. On the other hand, the conflict has precipitated a marked deceleration in global economic growth, heightened inflation, and elevated risks of a precipitous tightening in the global financing environment with capital outflows. This amplification accentuates global financial system risks and triggers the sustained fermentation of irrational factors, including investor sentiment [3]. The attributes of carbon emission rights encompass both resource allocation and financial functions [4], potentially exposing their prices to macro-level financial systemic risks and micro-level industry volatility shocks. Therefore, within the context of the Russia-Ukraine conflict, this study focuses on the transmission effects and the extent of the impact of systemic risk, energy market dynamics, and fluctuations in investor sentiment on carbon emissions trading prices. It is noteworthy that, as the direction and impact of the Russia-Ukraine conflict may evolve over time, its influence on relevant markets will also change, with varied concerns among different stakeholders. For example, traders may emphasize short-term price fluctuations, while policymakers may prioritize the long-term development of the market [5]. Across different periods, variations in the relationships and characteristics among these economic variables may exist. Clarifying these issues first and foremost contributes to safeguarding the interests of major participants in carbon market transactions. Secondly, it aids in identifying principles aligning with extreme events and inherent patterns between the Russia-Ukraine conflict and the Chinese carbon emissions trading market. This, in turn, advances the construction of the carbon market and the development of a low-carbon economy, ultimately providing crucial insights for mitigating systemic risks on a global scale.

The paper’s contribution can be outlined in two main aspects. Firstly, it focuses on the Russian-Ukrainian conflict and the Chinese carbon trading market, conducting an in-depth investigation into the internal mechanisms and transmission effects of the Russian-Ukrainian conflict on the Chinese carbon emissions trading market. This addresses existing research gaps, particularly the predominant focus on the mature European carbon trading market, neglecting the emerging market with a late start and weaker trading mechanisms. The aim is to enhance the research on the causality and economic consequences of major emergencies in the field of carbon asset pricing. Secondly, the paper comprehensively considers the connection between the carbon market and the energy market. It incorporates financial assets and micro-level investor sentiment into a systematic analytical framework, combining the complexity of financial theory and econometric methodology. The study adopts the intermediation model, the TVP-SV-VAR model, and wavelet theory to analyze the time variation of time series, multi-scale time-frequency characteristics, and the leading-lagging relationship under a unified framework. This approach aims to provide a systematic, comprehensive, and accurate portrayal of the real-world context while further expanding the framework of previous research on carbon asset pricing.

## Literature review and development of hypotheses

### Literature review

Carbon emissions trading rights, as a market mechanism designed to promote the global reduction of greenhouse gas emissions, essentially involve trading the privilege to emit carbon dioxide and other greenhouse gases as commodities. The origins of this concept are closely linked to the economist Pigou’s theory of "externalities," where the actions of individual economic entities impact the community or other sectors without incurring corresponding obligations or rewards. This idea is also influenced by Coase’s "property rights theory," which aims to minimize social costs by clarifying property rights related to environmental resources, allocating them through specific means, and enabling trading based on these rights. Existing literature on carbon emissions trading price research is primarily divided into areas such as factors influencing price volatility, characteristics of price volatility, and forecasts. This paper will mainly focus on organizing information from these two levels.

In terms of the factors influencing the price volatility of carbon emission rights trading, macroeconomics emerges as the most fundamental and long-term determinant impacting the carbon price in the EU [6]. However, without accounting for the enduring influence of technological progress, industrial upgrading, and other factors, short-term dynamics between the energy market (predominantly fueled by coal, natural gas, petroleum, and electricity) and the carbon market reveal a production inhibition effect, a substitution effect, and an aggregate demand effect [7]. Moreover, as the supply of carbon quotas is primarily allocated through the government’s unified quota allocation program, the volatility of carbon prices is significantly influenced by different quota plans, ratios, and inter-temporal reserve systems [8]. Simultaneously, with the evident trend of commodity financialization, the financial system has gradually become a crucial factor affecting carbon prices [9]. This impact operates on two fronts: influencing carbon prices through investors’ trading decisions via financial market information and playing a role in carbon prices through the liquidity path and exchange rate path. It is essential to note that, beyond the aforementioned factors, certain external, sudden, and unpredictable events also impact carbon prices, such as the financial crisis [10], the European debt crisis [11], and the COVID-19 pandemic [12]. Existing studies have explored the influencing factors of carbon prices from multiple perspectives, including macro economy, the energy market, economy and finance, and major emergencies. While this has laid a solid foundation for existing research, there are areas for improvement. Firstly, in the research on the carbon price of major emergencies, most previous literature examines the economic consequences of major emergencies affecting carbon prices but lacks sufficient focus on the causality of the research. Specifically, regarding the emerging Russian-Ukrainian conflict, existing studies predominantly concentrate on the quantitative analysis of prices in traditional energy and food industries [2, 13, 14], with limited exploration of carbon assets. Secondly, many studies on factors affecting carbon prices concentrate on specific elements, with fewer investigations combining and quantitatively analyzing various factors. Although some scholars have established the research framework of "carbon market-finance-energy" [15], there is a challenge in selecting comprehensive indicators when constructing proxy variables, which may not fully reflect the real situation. Additionally, within the current commodity pricing mechanism and the financialization of commodities, there is a lack of relevant research based on the behavior of micro subjects. Furthermore, the above research predominantly focuses on the mature EU market, with a shortage of studies on emerging countries, including China.

The research on the price volatility characteristics of carbon emission trading rights can be broadly categorized into two types of literature. The first type primarily focuses on studies at the levels of first-order and second-order moments of the price. Regarding the first-order moments, scholars typically employ VAR family models, considering time series returns. Examples include BVAR and the Diebold-Yilmaz spillover index [16, 17]. In terms of second-order moments, researchers predominantly investigate volatility measures, forecasts, and their correlations using GARCH family models, such as DCC-APGARCH, DCC-T-GARCH, DCC-GJR-GARCH models, and GARCH-MIDAS models [18, 19]. The second type of literature explores the subject from the perspective of time-frequency scale processing of time series, employing techniques like wavelet analysis and quantile models [20, 21]. These methods decompose series into various time and frequency components, providing diverse perspectives for estimation. However, there is a relative gap in the research of related techniques in the second type of literature, particularly within the field of carbon pricing. Thus, the well-established applications of these methods in other commodity fields [22, 23] offer valuable references for research related to the carbon asset field. This paper, building on the aforementioned literature, draws inspiration from the works of Meng et al. [24] and Hou et al. [18]. Recognizing the time-varying characteristics of the Russian-Ukrainian conflict and the potential limitations of traditional methods in capturing important time-varying information, we employ the TVP-SV-VAR model. This model comprehensively considers time-varying influence relationships, enabling not only the analysis of impulse responses between variables within the same time context but also the analysis of impulse responses between variables in different time contexts. This approach allows for a more accurate depiction of time-varying relationships. Simultaneously, due to the complex and variable correlation between the Russian-Ukrainian conflict and its transmission channels, we incorporate wavelet analysis to achieve multi-scale decomposition of a single time series. This method measures and analyzes the interactive influence of variables under different time and frequency cycles. By uncovering the interactive relationship between variables under dual time-frequency scales, we enhance the understanding of the general operational mechanisms and laws among the variables.

### Development of hypotheses

**Fig 1:**
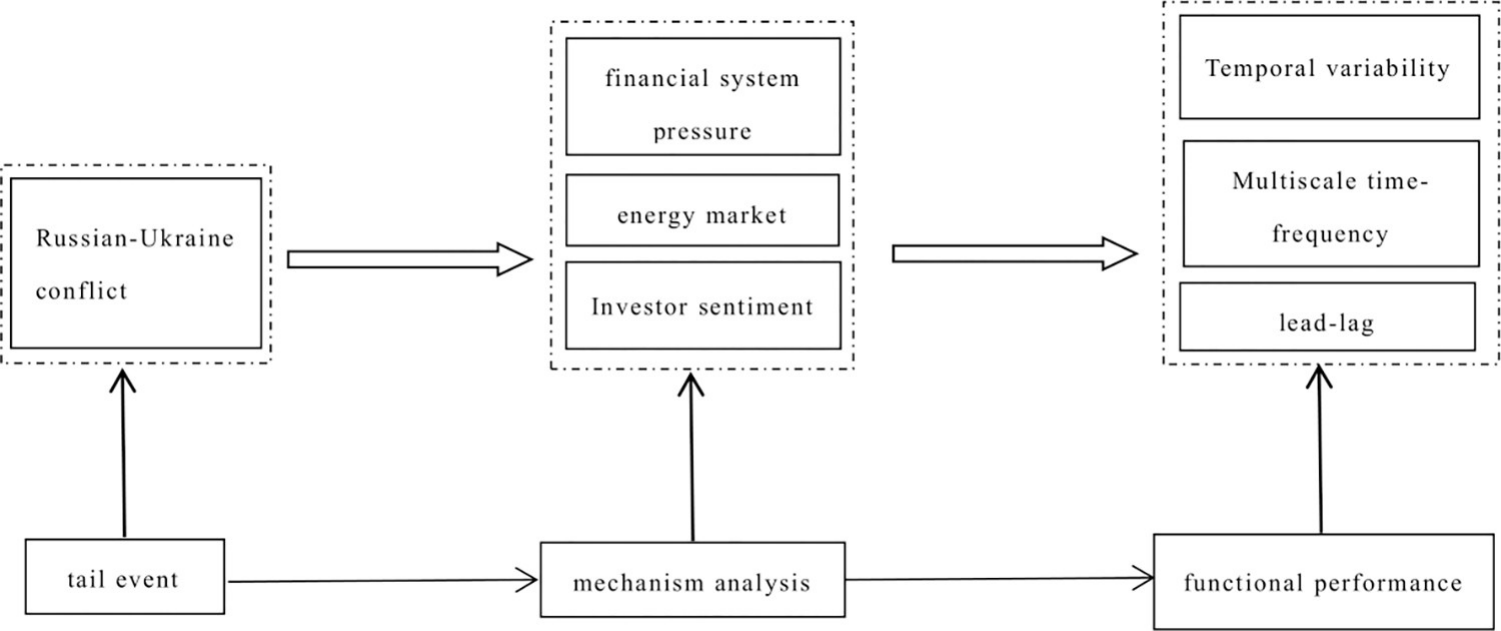
The transmission effects of the Russo-Ukrainian conflict on carbon emission trading prices.

This study investigates the transmission channels of carbon emission trading prices affected by the Russo-Ukrainian conflict, employing both theoretical frameworks and empirical analysis. These channels encompass pressures on the financial system, the energy market, and investor sentiment, as depicted in Fig 1.

#### 1. Financial system pressure

The Russo-Ukrainian conflict has precipitated various challenges, including heightened financial burdens and a deteriorating global trade environment. As major economies grapple with tough decisions involving interest rate hikes, balance sheet reductions, and inflation decreases, the uncertainties and complexities of policy adjustments intensify. When coupled with the commodity and financial dimensions of carbon emission trading, these factors can contribute to the transmission of adverse economic effects from the economy to the financial system and, ultimately, to the carbon market through capital flow pathways. The theory of the fourth-generation currency crisis highlights that capital flow is a pivotal catalyst for financial system turmoil [25]. Sudden external shocks to a financial institution or market can swiftly propagate throughout the system, compelling stakeholders to utilize various capital flow channels, such as stock and bond markets, to pursue capital gains and engage in exploitative behaviors, thereby inducing market fluctuations. Based on this analysis, this study posits hypothesis H1:

H1: The Russo-Ukrainian conflict significantly influences carbon emission trading prices through pressures on the financial system.

#### 2. Energy market

Russia holds a pivotal position in the energy market, with Europe importing approximately 37% and 30% of its natural gas and crude oil from Russia, respectively. Developing economies also rely on Russia, importing 25% of natural gas, 18% of coal, and 11% of crude oil. The Russo-Ukrainian conflict has induced substantial fluctuations in energy prices, disrupting global commodity production and service supply. The inherent interdependence between energy prices and carbon emission trading creates a linkage effect between the two markets. Fluctuations in energy prices not only affect actual energy demand in the real economy through income effects but also influence the preference for relatively lower-priced energy types through substitution effects. These combined factors impact carbon quota demand and contribute to price fluctuations. Building on this analysis, we propose hypothesis H2:

H2: The Russo-Ukrainian conflict significantly impacts carbon emission trading prices through energy market pressure.

#### 3. Investor sentiment

After the outbreak of the Russo-Ukrainian conflict, the speculative atmosphere surrounding commodities has heightened. With the escalation of the conflict, geopolitical tensions are on the rise, leading to an increase in global risk aversion. In accordance with Behavioral Finance Theory, the emergence of specific risks influences the psychology, behavior, and emotions of traders, impacting their trading decisions. Market traders, driven by rational expectations or irrational panic emotions, make decisions aimed at maximizing profits or adjusting their trades, both of which can induce fluctuations in asset prices. Building on this analysis, we propose hypothesis H3:

H3: The Russo-Ukrainian conflict significantly impacts carbon emission trading prices through investor sentiment.

Previous studies have indicated that investors and the market’s perception, as well as their response to extreme risks, may vary as events unfold [26]. Consequently, the financial market pressures, energy market, and investor sentiment considered in the theoretical analysis above might demonstrate changes in their impact on the carbon market throughout the evolution of the Russo-Ukrainian conflict. The Fractal Market Hypothesis posits that, owing to the presence of investors with diverse investment horizons, the market forms a specific fractal structure characterized by long-term memory, system mutation, sensitivity, and time-varying variance. Therefore, against the backdrop of the Russo-Ukrainian conflict, the transmission effects of its action channels on carbon emission trading prices may manifest differences, encompassing temporality, multi-scale time-frequency characteristics, and leading-lagging relationships. Based on this analysis, we propose hypothesis H4:

H4: In the context of the Russo-Ukrainian conflict, the impact of its action channels on carbon emission trading prices manifests in terms of temporality, multi-scale time-frequency characteristics, and leading-lagging relationships.

## Research design

### Model specification

The primary empirical approach in this study is outlined as follows: First, we establish a benchmark regression model to assess the significant impact of the Russo-Ukrainian conflict and its three major transmission channels on carbon emission trading prices. The model (1) is specified as follows:

$$P_{t}=\beta_{0}+cD_{t}+\beta_{2}{Controls}_{t}{+\varepsilon }_{t}$$ (1)

To examine the hypothesized mediating pathway, we additionally formulate mediation effect models building upon model (1):

$$M_{it}=c_{0}+aD_{t}+c_{2}{Controls}_{t}+\varepsilon_{t}$$ (2)

$$P_{t}=d_{0}+c^{\prime }D_{t}+bM_{it}+d_{3}{Controls}_{t}$$ (3)

Where P_t_ represents the dependent variable, which is the carbon emission trading price. D_t_ is the independent variable, a dummy variable representing the occurrence of the Russo-Ukrainian conflict. M_it_ represents the mediating variables, including investor sentiment (CICSI), financial system stress (CFSI), and energy market (EMSI). A series of control variables is included, such as the Keqiang Index, the Consumer Price Index, public expenditure on energy conservation and environmental protection, and the Air Quality Index of key cities. Eit represents the random error term. Since the coefficient product method effectively overcomes the limitations of stepwise regression, we adopt it and use bootstrap sampling to verify whether the Russo-Ukrainian conflict affects carbon emission trading prices through the three major transmission channels of financial system stress, energy market, and investor sentiment (H1-H3).

After confirming the significant impact of the Russo-Ukrainian conflict and transmission channels on carbon emission trading prices, we employ the TVP-SV-VAR model [27] and wavelet theory [28] to quantify the transmission effects (H4). Firstly, we use the TVP-SV-VAR model to verify the time-varying characteristics of the channel variables, and the model is specified as follows:

$$Y_{t}=X_{t}\beta_{t}+{A_{t}}^{-1}\Sigma_{t}\varepsilon_{t}$$ (4)

$$\beta_{t+1}=\beta_{t}+\mu_{\beta_{t}},\;\alpha_{t+1}=\alpha_{t}+\mu_{\beta_{t}},\;h_{t+1}=h_{t}+\mu_{\beta_{t}}$$ (5)

$$\left[\varepsilon_{t},\;\mu_{\beta_{t}},\;\mu_{\beta_{t}},\;\mu_{\beta_{t}}]∼[0,\;(I_{t},\;\Sigma_{\beta },\;\Sigma_{\alpha },\;\Sigma_{h})\right]$$ (6)

Where, Y_t_ includes the carbon emission trading price and the channel variables. $X_{t}=I_{t}(y_{t-1},y_{t-2},\ldots ,y_{t-s}),$ represents the Kronecker product, and I_t_ is the identity matrix. The coefficients β_t_, the joint parameter matrixA_t_, and the random fluctuation covariance matrix Σ_t_ all follow a random walk process. We use the Markov Chain Monte Carlo (MCMC) sampling method to obtain the actual results.

Next, we employ wavelet theory to verify the multi-scale time-frequency and lead-lag relationship of the channel variables. The specific steps are as follows: We use the Continuous Wavelet Transform to construct the orthogonal projection space and corresponding basis with different time series resolutions by scaling and translating the Morlet wavelet function. This process is represented as follows:

$$\Psi (t)=\pi^{-1/4}e^{iw_{0}t}e^{-t^{2}/2}$$ (7)

To achieve a balance between time and frequency localization, the parameter ω_0_ is typically set to 6. Additionally, we employ the Cross Wavelet Transform to capture the correlation between high-energy regions of the time series, while the Wavelet Coherence is used to capture the correlation between low-energy regions. Assuming that the channel variable is $X=\{x_{i}|i=1,2,\ldots ,n\}$ i and the carbon emission trading price is $Y=\{y_{i}|i=1,2,\ldots ,n\}$, they can be represented as:

$$w_{n}^{XY}(s)=w_{n}^{X}w_{n}^{Y*}(s)$$ (8)

$$R_{n}^{2}(n)=\frac{{\left|S(s^{-1}w_{n}^{XY}(s))\right|}^{2}}{S[s^{-1}{\left|w_{n}^{X}\left(s\right)\right|}^{2}]*S[s^{-1}{\left|w_{n}^{Y}\left(s\right)\right|}^{2}]}$$ (9)

$$\phi_{XY}(s)={tan}^{-1}\left\{\frac{s^{-1}w_{n}^{XY}(s)}{s^{-1}w_{n}^{XY}(s)}\right\}$$ (10)

In this context, $w_{n}^{xy}(s)$ represents the absolute value of the cross-wavelet power spectrum, which denotes the absolute value of energy resonance and covariance at a specific frequency. $R_{n}^{2}(n)$ is the wavelet coherence coefficient, which indicates the degree of dependence between two time seriesand ϕ_XY_(s) represents the phase difference, which denotes the oscillation phase relationship between two time series.

### Indicator setting

The article examines the following variables: (1) Dependent variable: Carbon Emission Trading Price (P_t_). According to data from the Chinese Ministry of Ecology and Environment, the Chinese carbon trading market covered about 4.5 billion tons of carbon emissions in the first compliance cycle year, making it the world’s largest carbon emissions trading market. Since previous studies mainly focused on the mature European carbon trading market, there is a scarcity of research on emerging markets, including China. Hence, it is crucial to prioritize the Chinese carbon trading market as the primary research focus to enhance the understanding of typical global carbon markets. It’s worth noting that China has established nine carbon emission rights pilots, but some of these markets face issues such as small trading volume and limited liquidity. Therefore, we select five carbon trading pilots in China—Hubei, Shenzhen, Guangdong, Beijing, and Shanghai—as sample data due to their robust turnover and liquidity of trading funds. To optimize the use of market information and leverage the comparative strengths and contrasts of the selected pilots, we employ the CRITIC method for assignment. The average price of emission right transactions in the five carbon markets is then synthesized into the comprehensive price of carbon emission rights.

(2) Independent variable: Indicator for the occurrence of the Russia-Ukraine conflict. When the sample is in the ith stage, the corresponding variable value is 1; otherwise, it is 0. On February 24, 2022, Russia officially launched a special military operation against Ukraine; thus, February 2022 was designated as the outbreak of the Russia-Ukraine conflict.

(3) Control variables: Following the method of Gao et al. [29], the designated control variables are as follows: ① Economic level, represented by the "Keqiang Index." This index monitors economic operations from three dimensions: power grid, railway, and bank. Compared to GDP, it offers greater practicality and authenticity. ② Consumption level, represented by the Consumer Price Index for residents; ③ Government environmental protection efforts, indicated by public financial expenditure on energy conservation and environmental protection. ④ Climate level, represented by the comprehensive air quality index of the cities where the five selected carbon trading pilots are located.

(4) Mediating variables: Investor sentiment (CICSI), financial system pressure (CFSI), and energy market (EMSI). For investor sentiment, the CICSI index established by Yi et al. [30] serves as an indicator of investor sentiment in the carbon emission trading market. The CICSI index includes six indicators measuring objective market sentiment, such as the fund discount rate and the number of companies going public for the first time each month. Regarding the financial system pressure index and energy market index, we draw on the studies of Li et al. [31] and Bibi et al. [32] to construct these indices. The construction process involves two steps: First, 12 and 7 basic indicators are selected as representatives of the financial system and energy sub-markets, respectively, as shown in Table 1. The pressure index of each sub-market is synthesized using the reciprocal of the standard deviation of the characteristic indicators as weights, expressed mathematically as follows:

ωi=1σi∑σj (11)

In the equation, ω_i_ represents the weight of the i-th indicator in the sub-market, while σ_i_ represents the standard deviation of the i-th indicator, indicating the amount of information contained in the data. The greater the standard deviation, the greater the weight. In the second step, the CRITIC weighting method is used to synthesize the pressure index of each sub-market into a system index, expressed mathematically as follows:

$$C_{i}=\sigma_{i}*(\sum \left(1-r_{ij}\right))$$ (12)

$$W_{i}=\frac{C_{i}}{\sum C_{i}}$$ (13)

Where, W_i_ represents the weight of the i-th market, and σ_i_ represents the standard deviation of the i-th market, indicating the correlation coefficient between market i and market j. ∑(1−r_ij_) represents the conflict between markets, with greater indicator conflict resulting in greater weight.

**Table 1:** Selection and calculation methods of structural variables for financial system stress index and energy market index.

Panel A: Financial System Stress Index			
Market	Indicator	Description	Direction
Money Market	TED Spread	The indicator is the difference between the 3-month SHIBOR and the 3-month fixed deposit rate, reflecting the liquidity situation in the money market.	Positive
	Weighted Average Interest Rate of 7-day Repo Agreements in the Interbank Market	The indicator reflects the short-term funding needs and liquidity situation in the money market.	Positive
Stock Market	Stock Market Growth Rate	The indicator is the growth rate of the Shanghai Composite Index (P1-P0)/P0, reflecting the price growth rate of the stock market.	Negative
	Stock Market Volatility	The indicator is the volatility of the Shanghai Composite Index, obtained through GARCH(1,1), reflecting the price volatility of the stock market.	Positive
Real Estate Market	Real Estate Market Growth Rate	The indicator is the growth rate of the National Real Estate Prosperity Index (P1-P0)/P0, reflecting the growth rate of the real estate market.	Negative
	Real Estate Market Volatility	The indicator is the volatility of the National Real Estate Prosperity Index, obtained through GARCH(1,1), reflecting the volatility of the real estate market.	Positive
Bond Market	Term Spread	The indicator is the difference between the yield of 5-year and 1-year government bonds, reflecting the term spread in the bond market.	Positive
	Bond Market Volatility	The indicator is the volatility of the logarithmic yield of 1-year and 5-year bonds, obtained through equally weighted GARCH(1,1), reflecting the volatility of the bond market.	Positive
Foreign Exchange Market	Exchange Rate Market Growth Rate	This indicator is the percentage change in the real effective exchange rate of the Chinese yuan (P1-P0)/P0, reflecting the growth rate of the exchange rate market.	Negative
	Exchange Rate Market Volatility	This indicator is the volatility of the index of the real effective exchange rate of the Chinese yuan, obtained through GARCH (1,1), reflecting the volatility of the exchange rate market.	Positive
External Market	TED Spread	This indicator is the difference between the 3-month London Interbank Offered Rate (LIBOR) and the 3-month US Treasury Bill yield, reflecting the short-term liquidity of the international financial market.	Positive
	Dow Jones Industrial Average Volatility	This indicator is the volatility of the Dow Jones Industrial Average, obtained through GARCH (1,1), reflecting the volatility of the international financial market.	Positive
Panel B: Energy Market Index			
Crude Oil Market	MICEX Brent Crude Oil Active Contract Closing Price Volatility	This indicator represents the volatility of the closing price of the MICEX Brent Crude Oil Active Contract, obtained through GARCH(1,1), reflecting the volatility of overseas crude oil prices.	Positive
	China Daqing Crude Oil Spot Price Volatility	This indicator represents the volatility of the China Daqing Crude Oil Spot Price, obtained through GARCH(1,1), reflecting the growth rate of crude oil prices in the Chinese market.	Positive
Natural Gas Market	NYMEX Natural Gas Active Contract Closing Price Volatility	This indicator represents the volatility of the closing price of the NYMEX Natural Gas Active Contract, obtained through GARCH(1,1), reflecting the volatility of overseas natural gas prices.	Positive
	China Natural Gas and Synthetic Gas Import Price Index Volatility	This indicator represents the volatility of the China Natural Gas and Synthetic Gas Import Price Index, obtained through GARCH(1,1), reflecting the volatility of natural gas prices in the Chinese market.	Positive
Coal Market	IPE Rotterdam Coal Active Contract Closing Price Volatility	This indicator represents the volatility of the closing price of the IPE Rotterdam Coal Active Contract, obtained through GARCH(1,1), reflecting the volatility of overseas coal prices.	Positive
	China Coke Futures Active Contract Closing Price Volatility	This indicator represents the volatility of the closing price of the Coke Futures Active Contract, obtained through GARCH(1,1), reflecting the volatility of coal prices in the Chinese market.	Positive
Carbon Quota Market	EU Emissions Allowance (EUA) Volatility	This indicator represents the volatility of the closing price of the EU Emissions Allowance (EUA), obtained through GARCH(1,1), reflecting the volatility of overseas carbon quota prices.	Positive

Note: P1 represents the mean value for the current month, while P0 represents the mean value for the previous month.

### Data source

In this paper, the data’s initial period is designated as January 2015, with a cut-off period until December 2022. This timeframe is primarily guided by practical considerations, given the disparity in the online trading commencement of the five selected carbon trading pilots in China—Hubei, Shenzhen, Guangdong, Beijing, and Shanghai. To establish a consistent starting point for the examination, the date of the online trading initiation of the Hubei market, the latest to commence online trading (February 2014), is chosen. Upon further investigation, it was observed that the overall trading volume was limited and unstable during the initial phase of its carbon trading market. Considering the main focus of this paper on the impact of the Russo-Ukrainian conflict on carbon prices, the initial data period is further adjusted, setting it to January 2015. Additionally, the time window undergoes further processing in the robustness test section of the benchmark regression. The data utilized in this study are sourced from the WIND database, and the following pre-processing steps are applied: (1) Transformation of daily frequency benchmark data into monthly frequency data through arithmetic averaging; (2) Handling of missing data using linear interpolation; (3) Execution of X-12 seasonal adjustment and normalization, with each series verified to pass the ADF test.

## Results analysis

### Baseline regression results

Using model (1), the ordinary least squares (OLS) method was applied to assess the impact of the Russia-Ukraine conflict (Dt) on carbon emission trading prices (Pt), with estimated robust standard errors. The results are presented in Table 2. Columns (1), excluding control variables, and (2), incorporating relevant control variables, reveal that the dummy variable representing the Russia-Ukraine conflict has a statistically significant positive impact on carbon emission trading prices at the 1% significance level. This suggests that the conflict positively affects carbon emission trading prices, supporting H1. The Russia-Ukraine conflict disrupts the global energy market supply, resulting in an increase in Chinese carbon emission trading prices due to its energy demand and consumption intensity. Furthermore, given the interconnected nature of the energy and carbon markets, fluctuations in the energy market can trigger a chain reaction in the carbon market, even within China. Therefore, the delayed initiation and lower degree of integration of the Chinese carbon market with external factors compared to overseas markets do not shield it from the repercussions of the Russia-Ukraine conflict on carbon emission trading prices.

**Table 2:** Benchmark regression results.

	P_t_	P_t_
D_t_	0.545*** (8.09)	0.438*** (6.13)
controls		YES
Constant	0.153*** (14.23)	0.425*** (6.42)
R^2^	0.6865	0.7583
F-value	65.50	11.8

Note

* p<0.10

** p<0.05

*** p<0.01. The values in parentheses represent the t-values.

### Robustness test

To enhance the reliability of the benchmark model’s conclusions, three additional methods were employed to substantiate the main research findings in this paper. These methods involve shortening the window period, lagging the dependent variable by one period, and replacing the dependent variable.

1. The window period was reduced from 8 years to January 2020-December 2022, as an extended window period may attenuate the impact of the Russia-Ukraine conflict on carbon emission trading prices. The results, presented in Table 3, column (1), reveal that the research conclusion remains significant, with Pt showing a significant positive effect at the 1% level.
2. Considering the lag effect of the Russia-Ukraine conflict on Chinese carbon emission trading prices, the dependent variable was lagged by one period. Table 3, column (2), illustrates that the research conclusion remains valid, with Pt continuing to exhibit a significant positive impact at the 1% level.
3. The dependent variable was substituted with carbon trading volume to explore the negative correlation between carbon price and trading volume. The comprehensive carbon emission trading volume Q_t_ was constructed using the CRITIC method to assign weights to the carbon trading volumes of five carbon trading pilot projects. Column (3) of Table 3 confirms the significant negative correlation between the Russia-Ukraine conflict and carbon trading volume, indirectly suggesting a positive correlation between the conflict and carbon price.

**Table 3:** Robustness test results.

	P_t_	P_t_	Q_t_
D_t_	0.387*** (5.09)	0.411*** (5.83)	-0.197*** (-3.16)
controls	YES	YES	YES
Constant	0.677*** (3.69)	0.375*** (5.38)	0.470*** (3.39)
R^2^	0.7654	0.7297	0.1316
F-value	7.84	9.65	1.97

Note

* p<0.10

** p<0.05

*** p<0.01. The values in parentheses represent the t-values.

### Mechanism analysis

Building on the earlier analysis suggesting that the Russia-Ukraine conflict impacts carbon emission trading prices, a mechanism analysis was conducted to further examine the influence of major conflict events using the Bootstrap method and an intermediary effect testing procedure, as explained in the preceding section. The results presented in Table 4 reveal significant intermediary effects for both financial market pressure (CFSI) and energy market pressure (EMSI), with confidence intervals of (0.028 ~ 0.191) and (0.009 ~ 0.389), constituting 14.662% and 26.487%, respectively. Both intervals exclude 0, indicating the active role of these intermediary variables. In contrast, investor sentiment did not pass the significance test, suggesting an absence of an intermediary effect. Moreover, the direct effect demonstrates a positive and significant coefficient of 0.289 at the 5% level, with a confidence interval of (0.130 ~ 0.448), signifying a partial intermediary role for the two variables.

**Table 4:** Results of bootstrap mediation test.

Mediation Effect Test	Russia-Ukraine Conflict = >CFSI = >P	Russia-Ukraine Conflict = > EMSI = >P	Russia-Ukraine Conflict = >CICSI = >P
a	-0.251**	0.483**	-0.237**
b	-0.256**	0.240*	0.132
Mediation Effect(a*b)	0.064	0.116	-0.031
Interval of Mediation Pathway Effect	0.028 ~ 0.191	0.009 ~ 0.389	-0.111 ~ 0.019
Direct Effect (*c*′)	0.289**	0.289**	0.289**
Interval of Direct Effect	0.130 ~ 0.448	0.130 ~ 0.448	0.130 ~ 0.448
Direct Effect/Indirect Effect	14.662%	26.487%	0%
Test Results	Partial Mediation	Partial Mediation	Non-Significant Mediation Effect

Note

* p<0.10

** p<0.05

*** p<0.01. The values in parentheses represent the t-values.

In summary, financial market pressure and energy market pressure function as intermediaries, indicating a connection between the Russia-Ukraine conflict, these factors, and the Chinese carbon market. Conversely, investor sentiment does not act as an intermediary, highlighting that macroeconomic factors exert a more substantial influence on the fluctuations of the Chinese carbon emission trading market. This can be attributed to two primary reasons. Firstly, the CICSI index reflects sentiment in the stock market, while the carbon market primarily involves physical enterprises, resulting in structural differences between the two markets. Secondly, during special events, investors tend to favor assets with high liquidity and substantial funds for speculative trading to enhance their success rate. The Chinese carbon emission trading market, characterized by limited arbitrage opportunities, insufficient liquidity, and trade primarily driven by task completion, is less susceptible to significant effects from changes in stock market investor sentiment.

## Further analysis

### TVP-SV-VAR model

**Fig 2:**
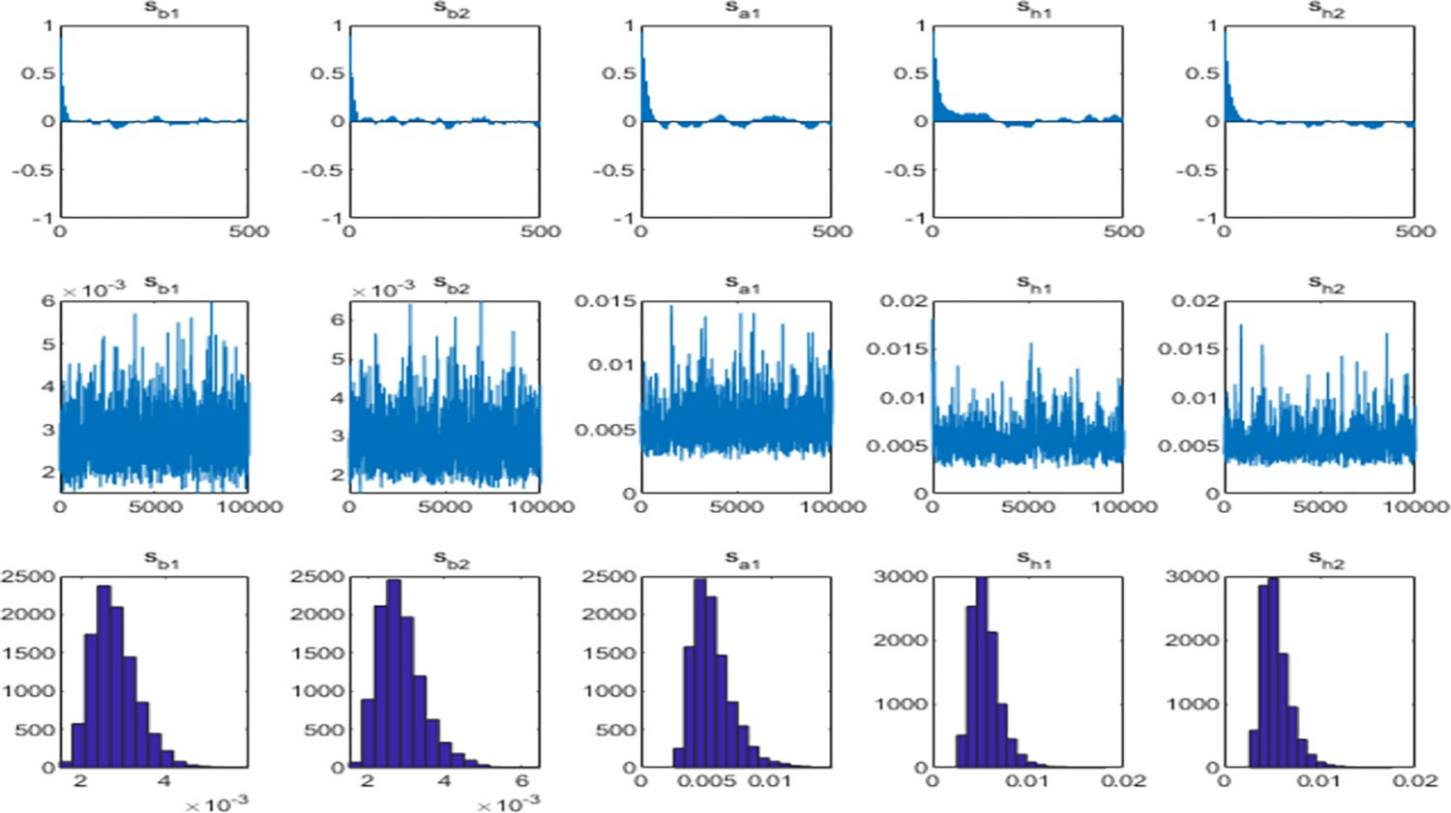
The parameter estimation results of the TVP-SV-VAR model.

Building on the preceding analysis, it becomes evident that financial system pressure (CFSI) and energy market pressure (EMSI) assume intermediary roles, whereas investor sentiment (CICSI) does not. Consequently, further investigation was carried out, focusing on the mechanisms of financial market pressure and energy market pressure, with the exclusion of investor sentiment. The optimal lag order for the VAR model was determined to be 1, as indicated by the minimum BIC and HQIC values. Subsequently, a Monte Carlo simulation was executed, involving 10,000 samples and a variable order encompassing carbon emission trading price, financial system pressure, and energy market. The results of the parameter estimation are detailed in Table 5 and illustrated in Fig 2. The parameter estimation results reveal that the mean of the posterior distribution falls within the 95% confidence interval, Geweke values are less than or equal to 1.96, and ineffective factors are fewer than 100. These outcomes suggest that the lag 1 order TVP-SV-VAR model can generate effective samples.

**Table 5:** Estimation results of parameters.

Parameter	Mean	Standard Deviation	95% Confidence Interval	Geweke Value	Ineffective Factor
s_b1_	0.0028	0.0005	[0.0020,0.0040]	0.000	12.27
s_b2_	0.0029	0.0006	[0.0020,0.0043]	0.698	19.08
s_a1_	0.0055	0.0016	[0.0033,0.0094]	0.000	19.68
s_h1_	0.0056	0.0016	[0.0034,0.0094]	0.334	41.64
s_h2_	0.0055	0.0015	[0.0034, 0.0093]	0.490	24.17

**Fig 3:**
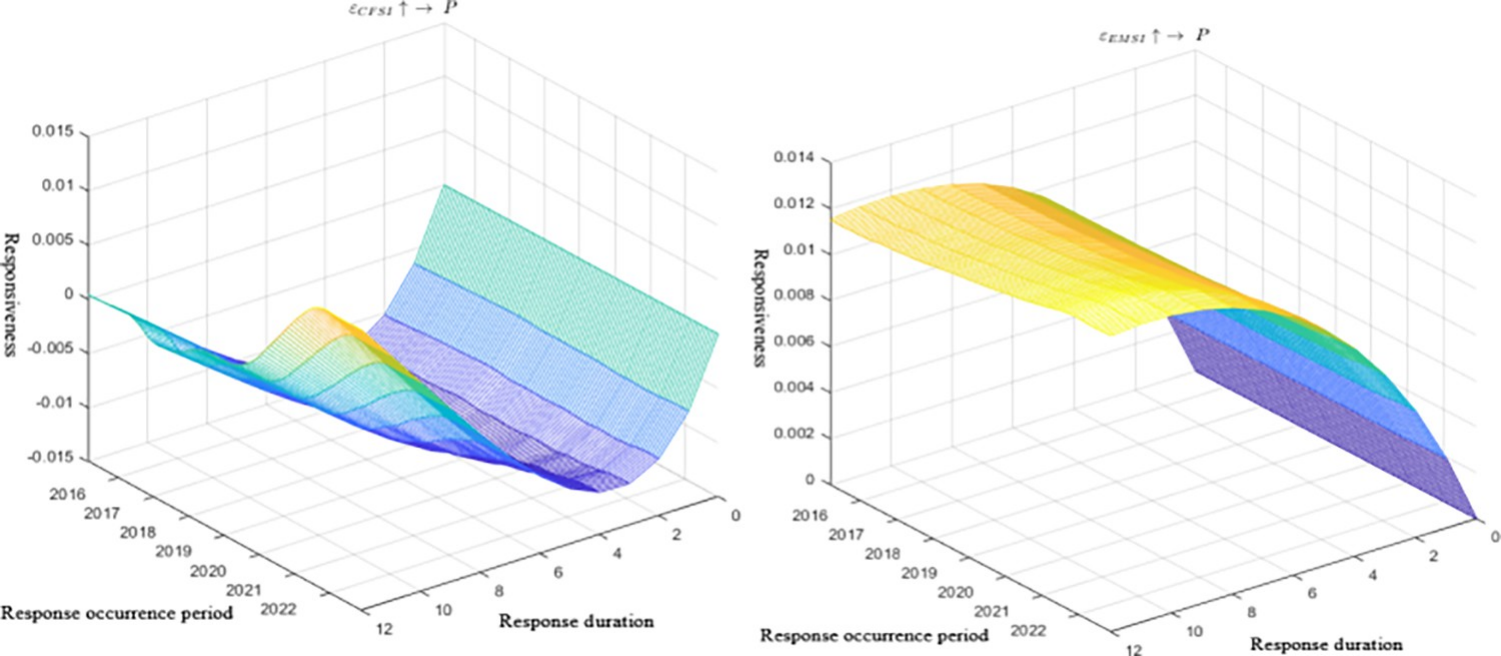
Three-dimensional pulse response graph.

**Fig 4:**
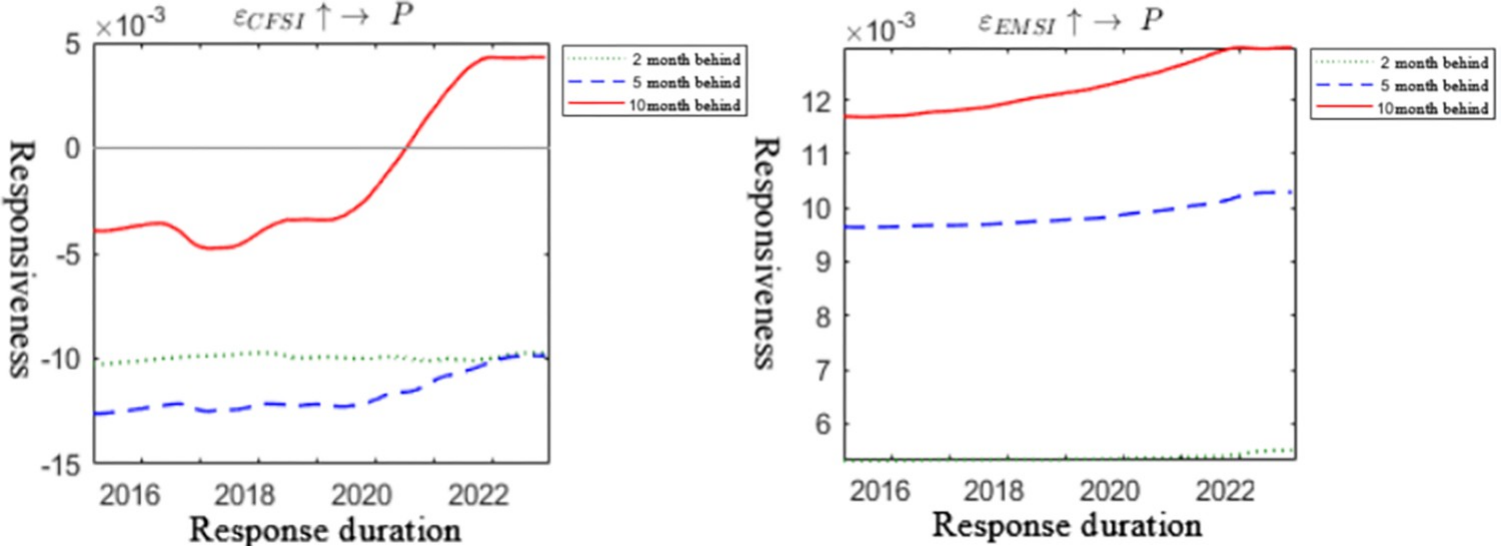
Two-dimensional pulse response graph.

**Fig 5:**
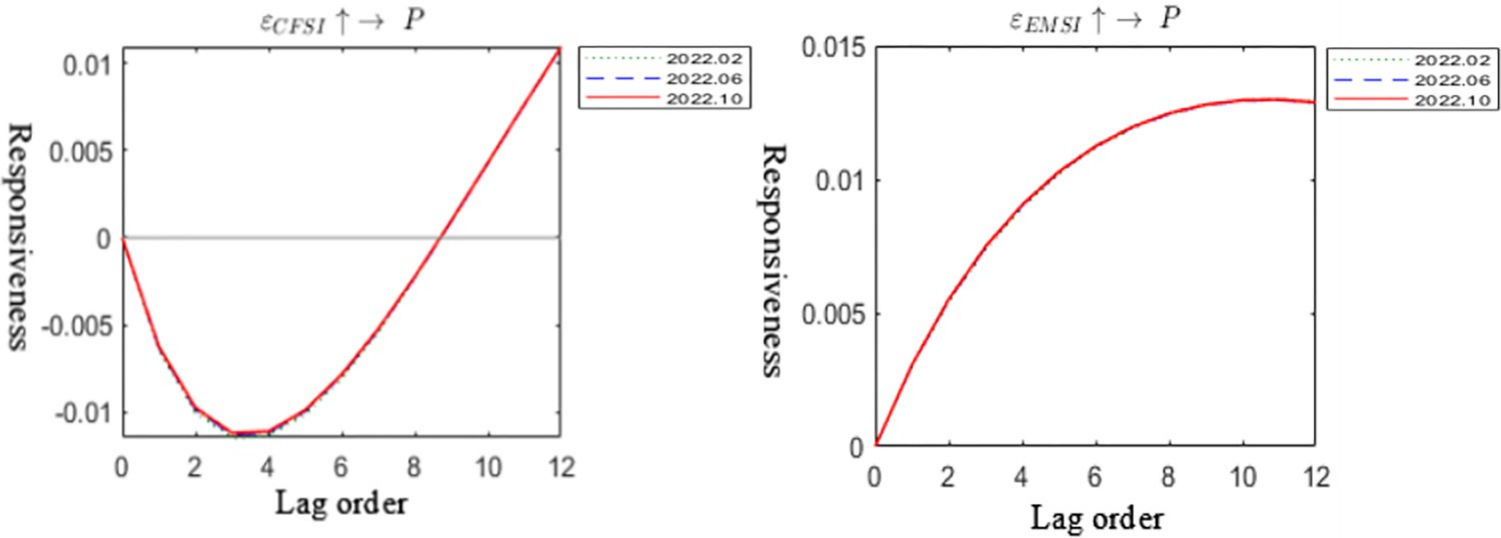
Special timing impact graph.

The three-dimensional impulse response graph in Fig 3 depicts the impact of financial system pressure and energy market on carbon emission trading prices, with axes representing the occurrence period, duration, and magnitude of the response. In Fig 4, a two-dimensional projection of the impulse response is presented, highlighting lag periods of 2, 5, and 10 intervals. Additionally, Fig 5 provides the impulse response graph at specific time points—March, June, and October 2022. These time points were chosen to capture the temporal characteristics of the Russia-Ukraine conflict’s influence on carbon emission trading prices during the early, middle (military conflict and comprehensive economic sanctions), and late stages of the conflict.

Figs 3 and 4 illustrates that financial system pressure and the energy market continuously influenced carbon emission trading prices from 2015 to 2022, even after the onset of the Russia-Ukraine conflict, supporting Hypothesis 1. Notably, the slope of the response of carbon emission trading prices to financial system pressure and the energy market has significantly increased since 2020, indicating that recent unforeseen events, such as the pandemic and the Russia-Ukraine conflict, have acted as "accelerators" and "boosters," significantly amplifying the linkage between financial system pressure, the energy market, and carbon emission trading prices. Fig 5 indicates that at three time points in February, June, and October 2022, the response of carbon emission trading prices to financial system pressure displays a U-shaped trend, while the response to energy system pressure shows a cumulative positive response, peaking after an 8-period lag. These findings suggest that under the backdrop of the Russia-Ukraine conflict, the transmission channel of carbon emission trading prices exhibits time-varying characteristics, preliminarily confirming Hypothesis 4.

From a channel perspective, the short-term response of carbon emission trading prices to financial system pressure follows a U-shaped trend after the onset of the Russia-Ukraine conflict, with negative transmission effects on carbon emission trading prices. However, in the long run, financial system pressure can provide positive support for carbon emission trading prices. This observation aligns with real-world events, as several developed economies implemented a three-pronged financial sanction approach against Russia post-conflict outbreak. These measures included the issuance of a special designated national list (SDN list) targeting major Russian banks, excluding some Russian banks from SWIFT, and freezing the foreign exchange reserves of the Central Bank of Russia. Consequently, bank runs, rising interest rates, falling stock markets, depreciation of the ruble, and a downgrade of Russia’s sovereign credit rating occurred, causing substantial impacts on the financial services entity economy and the cost of Russia’s international trade and investment activities. On one hand, the market expressed concerns over deteriorating US-China relations and potential decoupling risks between China and the US, leading to sell-offs of Chinese assets, including carbon emission trading prices, in the short term. This resulted in short-term negative impacts on several asset prices. However, as the situation progressed, Russia implemented measures such as raising key interest rates to 20% and prohibiting foreign exchange provisions overseas, which yielded certain effects. Additionally, the resilience of the Chinese economy and unchanged positive fundamentals caused liquidity funds to flow back into various assets, including carbon emission trading prices, providing positive support during later stages of financial system pressure. On the other hand, the response of carbon emission trading prices to the energy market is predominantly positive in the medium to long term, with less pronounced short-term effects. This conclusion partially explains the economic fact that amidst the Russia-Ukraine conflict, the EU carbon quota price sharply declined in the short term, while the Chinese carbon emission trading price remained strong, indicating that the energy market had little influence on the Chinese carbon emission trading price in the short term. During the later stages of the conflict, policy measures were introduced to address the impact of the conflict on the global energy market supply and demand, promoting carbon market reform and restarting coal-fired power plants in many countries. Additionally, energy prices surged, and the market used expectations of production reduction and supply tightening to speculate, resulting in increased profits for micro-energy industries and increased output for energy companies. Therefore, under macro policies and micro demand expectations, the established carbon quota allocation system in China will drive market anticipation for carbon quota demand and further raise carbon emission trading prices, providing support.

### Wavelet analysis

The cross-wavelet spectrum, wavelet coherence, and continuous wavelet transform were employed to analyze the relationships among financial system pressure (CFSI), energy market (EMSI), and carbon emission trading price (P) in the time-frequency scale. This method broadens the investigation into the mutual influence mechanisms of these variables. In the figure, the U-shaped black solid line illustrates the cone of influence, representing the wavelet boundary effect. The area enclosed by the thick black solid line depicts the Monte Carlo simulation estimation using phase randomization, replacing the sequence with a red noise test at a 95% confidence level. The horizontal axis signifies time, while the left axis denotes frequency. It is important to note that frequency and wave period have an inverse relationship; lower frequency corresponds to longer wave periods. The color bar on the right axis indicates the relative change in energy density. This comprehensive approach enhances our understanding of the dynamics between financial system pressure, energy market, and carbon emission trading prices across different time and frequency scales.

**Fig 6:**
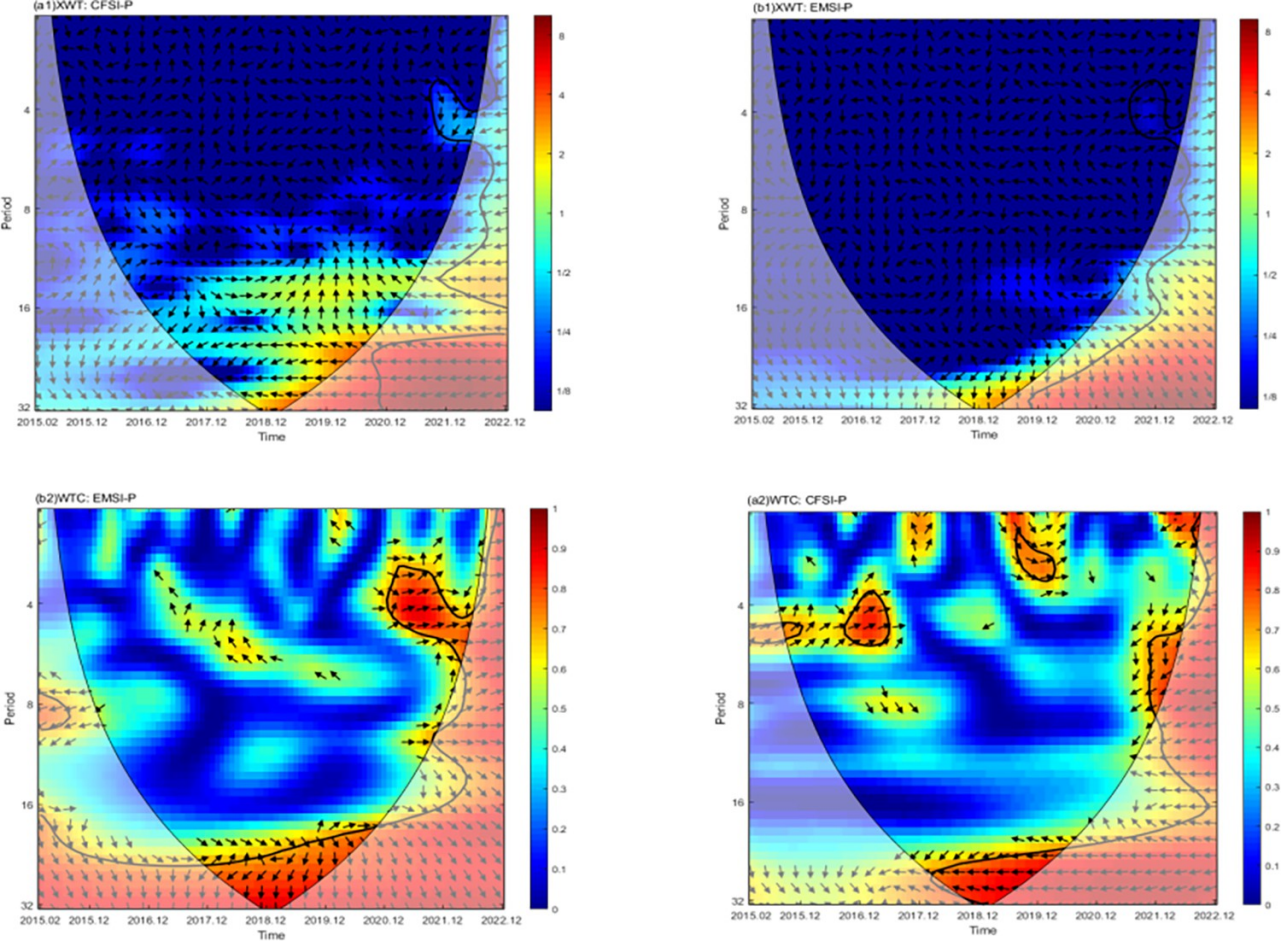
The Cross Wavelet and wavelet coherence analysis of financial system pressure, energy market pressure, and carbon emission trading price.

Fig 6 illustrates that following the outbreak of the Russia-Ukraine conflict, notable resonance regions emerge in the high-energy zone near the 4-month frequency scale and the low-energy zone near the 4–8 month frequency scale for both financial system pressure and carbon emission trading price (depicted in Fig 6(A1) and 6(A2)). This underscores a robust interdependence between financial system pressure and carbon emission trading price within the frequency scale of 0–8 months. Additionally, a significant resonance region appears in the low-energy area, close to the 4–8 month frequency scale, for energy market pressure and carbon emission trading price (as shown in Fig 6(B1) and 6(B2)), indicating a pronounced interdependence between energy market pressure and carbon emission trading price at the frequency scale of 4–8 months.

Furthermore, the overall direction of arrows for financial system pressure and carbon emission trading price points towards the lower left, signifying that changes in carbon emission trading price align with those in financial system pressure. Conversely, the overall direction of arrows for energy market pressure and carbon emission trading price points towards the right, suggesting a positive correlation between the two markets, consistent with the preceding analysis.

The above analysis verifies the presence of multi-scale characteristics and lead-lag dynamics in the time-frequency scale of financial system pressure, energy market, and carbon emissions trading price, confirming hypothesis 4. This conclusion implies that the financial system and the energy market hold pivotal roles in the carbon emissions trading market, exerting economic influence on changes in the price of carbon emissions trading. It serves as a reminder to stakeholders to attend to the dynamics of the financial system and the energy market in the decision-making process. Specifically, in the short- and medium-term, attention should be focused on the financial system market, closely linked to the carbon emissions trading market. In the medium- and long-term, vigilance should be exercised against the impacts of the energy market on the carbon emissions trading market.

## Conclusion

In this study, we employ the mediation test model, TVP-SV-VAR model, and wavelet analysis to investigate the transmission effects among financial system stress, the energy market, and Chinese carbon emission rights trading prices during the Russia-Ukraine conflict. First, our empirical findings unequivocally demonstrate that the onset of the Russia-Ukraine conflict impacts carbon emission rights prices through both financial market pressure and the energy market. However, the influence of investor sentiment is deemed insignificant. This observation indicates that the volatility of the Chinese carbon emission rights market, within the context of the Russia-Ukraine conflict, is more driven by macro-level factors than the behaviors of individual market participants. Secondly, post the Russia-Ukraine conflict, the response of carbon emission rights trading prices to the energy market exhibits a positive trajectory, peaking in the medium and long term. Conversely, the response of carbon emission rights trading prices to financial system pressure displays a U-shaped pattern, maintaining a positive impact on prices in the medium and long term. This suggests that, in comparison with the energy crisis amid the Russia-Ukraine conflict, the financial system pressure triggered by the conflict warrants attention. It plays a crucial role in early warning for systemic financial risks and risk management. Finally, through wavelet analysis, we confirm that the aforementioned variables exhibit multi-scale characteristics and lead-lag dynamics in the time-frequency scale. Specifically, the financial market pressure and the energy market contribute economically to driving changes in carbon emissions trading prices. This underscores the importance for stakeholders to promptly consider the dynamics of the financial system and the energy market in the decision-making process.

Based on the aforementioned conclusions, this paper proposes two policy recommendations. (1) it is crucial to effectively monitor and prevent relevant risks. Given the delayed inception of the Chinese carbon market, characterized by an incomplete market system, poor timeliness in information monitoring, and low accuracy, the government’s policy intervention often falls short in preventing problems before they manifest during major emergencies such as the Russia-Ukraine shock. Consequently, there is a need to enhance the early warning capability for carbon price risks stemming from major emergencies. This involves strengthening multi-party cooperation among the government, enterprises, and international organizations, improving information transparency, and establishing a coordinated early warning mechanism. This approach enables a prompt response to stabilize the carbon market when major emergencies occur. Simultaneously, it is imperative to establish a comprehensive monitoring system to oversee the carbon market and related risks. Collecting, analyzing, and reporting data related to carbon prices, with a focus on financial system data, are recommended. Emphasis should be placed on the financial system market and the energy market to timely detect and accurately assess the potential impact of major emergencies on carbon prices.(2) the focus should be on implementing precise and timely interventions. Since the Russia-Ukraine conflict primarily affects the carbon emissions trading market through the financial system and the energy market, the government needs to ensure the continuity, stability, and sustainability of macroeconomic policies and multiple subsystems of the financial system. Learning from the experience of the EU’s carbon market construction and aligning it with China’s developmental context, appropriate macroeconomic adjustment policies should be formulated according to different interactions at different times. This proactive approach aims to prevent systemic risks from adversely affecting carbon prices. The ongoing impact of the Russia-Ukraine conflict on the world’s energy pattern, while less severe for China than Europe, serves as a "wake-up call." Despite the short-term impact on the carbon market not being evident during energy market fluctuations, precautionary measures should be taken against long-term effects. Thus, to address energy shortages caused by geopolitical conflicts and other factors, China needs to actively broaden its energy import channels, reserve a sufficient amount of energy, and promote the transformation of the energy structure towards a cleaner and less dependent one on external energy sources.

This study suggests potential avenues for future research. For instance, exploring the repercussions of changes in European energy policy and international climate agreements triggered by the Russia-Ukraine conflict on carbon emissions trading prices is an area that warrants further investigation. Understanding the dynamics of these factors and their impact on the carbon market could contribute to a more nuanced comprehension of the causal links and economic consequences of the Russian-Ukrainian conflict on the carbon emissions trading market. Addressing these questions would represent a promising path for future research in this field.

## Supporting information

S1 File (ZIP)
