# Expression of Concern: Source tracing and contagion measurement of carbon emission trading price fluctuation in China from the perspective of major emergencies

**DOI:** 10.1371/journal.pone.0337273

**Published:** 2025-11-24

**Authors:** The PLOS One Editors

After this article [1] was published, the *PLOS One* Editors identified concerns about the article’s peer review.

In addition, the specific datasets and sources of data used in this study are not described in sufficient detail to be reproducible. The authors have not responded to requests for this information or to provide the underlying data in an accessible format for editorial review. Therefore, the *PLOS One* Editors issue this Expression of Concern as this article [1] does not comply with the PLOS Data Availability policy.

We regret that the issues were not addressed prior to the article’s publication.
