# Supplementary material for: Source tracing and contagion measurement of carbon emission trading price fluctuation in China from the perspective of major emergencies
Source: PLoS One. 2024 Mar 8;19(3):e0298811. doi: 10.1371/journal.pone.0298811 (PMC10923469; doi:10.1371/journal.pone.0298811)
Supplement: S1 File — (ZIP) [file pone.0298811.s001.zip › supporting information files/tvp-sv-var/tvpvar_m.pdf]

# Time-varying parameter VAR model using TVP-VAR package

Jouchi Nakajima

jouchi.nakajima@gmail.com

May 1, 2020

## 1 Introduction

**TVP-VAR** (Time-Varying Parameter Vector AutoRegression) is the package for a Bayesian analysis of the time-varying parameter VAR models. It implements the Markov chain Monte Carlo (MCMC) algorithm to generate samples from the posterior distribution of the TVP-VAR models. The TVP-VAR class is written in MATLAB, and can be used by linking the TVP-VAR package in MATLAB source codes. *You may use and modify this code at your own risk. Please cite Nakajima (2011) in products involving the application of TVP-VAR package.*

### 1.1 Running TVP-VAR

To use the TVP-VAR package, put the files of `TVPVAR` folder into the folder of the current directory where you are working. Alternatively, you can use the package by adding the `TVPVAR` folder to the working directory in MATLAB.

### 1.2 Files in TVP-VAR

The main files in TVP-VAR package are

- `setvar.m` – sets variables in the model;
- `mcmc.m` – estimates the model by MCMC algorithm;
- `TVPVAR-Package.m` – description of functions;
- `tvpvar_m.pdf` – this document;

with sample programs:

- `tvpvar_ex*.m` – example for use of TVP-VAR package;
- `tvpvar_ex.xlsx` – example data for `tvpvar_ex*.m`.

The package requires the following m-files:

- `ssmooth.m` – simulation smoother;
- `svsamp.m` – multi-move sampler for stochastic volatility;
- `impulse.m`, `drawimp.m` – time-varying impulse response;
- `ftsvar.m` – time-series variance;
- `fGeweke.m` – Geweke's statistics for convergence check;
- `fAt.m`, `fXt.m`, `fXh.m`, `fminv.m` – functions for matrices.

# 2 Implementation with TVP-VAR package

## 2.1 Model formulation and creating objects

In the TVP-VAR package, we use the representation of the time-varying parameter VAR model given by

$$y_t = c_t + B_{1t}y_{t-1} + \cdots + B_{st}y_{t-s} + e_t, \quad e_t \sim N(0, \Omega_t),$$

for $t = s+1, \ldots, n$, where $y_t$ is a $(k \times 1)$ vector of observed variables, $B_{1t}, \ldots, B_{st}$ are $(k \times k)$ matrices of time-varying coefficients, and $\Omega_t$ is a $(k \times k)$ time-varying covariance matrix. A recursive identification is assumed by the decomposition $\Omega_t = A_t^{-1}\Sigma_t\Sigma_t A_t^{\prime -1}$, where $A_t$ is a lower-triangular matrix with the diagonal elements equal to one, and $\Sigma_t = \mathrm{diag}(\sigma_{1t}, \ldots, \sigma_{kt})$. Define $\beta_t$ is the stacked row vector of $B_{1t}, \ldots, B_{st}$; $a_t = (a_{1t}, \ldots, a_{qt})'$ is the stacked row vector of the free lower-triangular elements of $A_t$; and $h_t = (h_{1t}, \ldots, h_{kt})$ where $h_{it} = \log \sigma_{it}^2$. The time-varying parameters follow the random walk process:

$$\begin{aligned} \beta_{t+1} &= \beta_t + u_{\beta t}, \\ a_{t+1} &= a_t + u_{at}, \\ h_{t+1} &= h_t + u_{ht}, \end{aligned} \qquad \begin{pmatrix} \varepsilon_t \\ u_{\beta t} \\ u_{at} \\ u_{ht} \end{pmatrix} \sim N\left(0, \begin{pmatrix} I & O & O & O \\ O & \Sigma_\beta & O & O \\ O & O & \Sigma_a & O \\ O & O & O & \Sigma_h \end{pmatrix}\right),$$

for $t = s+1, \ldots, n$, with $e_t = A_t^{-1}\Sigma_t\varepsilon_t$, where $\Sigma_a$ and $\Sigma_h$ are diagonal, $\beta_{s+1} \sim N(\mu_{\beta_0}, \Sigma_{\beta_0})$, $a_{s+1} \sim N(\mu_{a_0}, \Sigma_{a_0})$ and $h_{s+1} \sim N(\mu_{h_0}, \Sigma_{h_0})$. See Nakajima (2011) for the details.

The TVP-VAR package supports several types of the model specification. The following steps are involved in the model formulation:

- Use `setvar('data', vy, asvar, nlag)` to load $y = (y_1, \ldots, y_n)'$, and to set the names of variables (`asvar`) and the number of lags (`nlag`).
- (Optional) If you add the time-varying intercept $c_t$, write `setvar('intercept', 1)`.
- (Optional) If you assume non-diagonal covariance matrix for $\Sigma_\beta$, write `setvar('SigB', 1)`.
- Code `mcmc(N)` to implement the MCMC algorithm with $N$ iterations.
- (Optional) Code `drawimp(vt, fl)` to draw time-varying impulse response (see below).

## 2.2 Time-varying impulse response

The TVP-VAR package computes impulse response at each time point based on the average size of the stochastic volatility across the entire sample periods. The code `drawimp(vt, fl)` outputs graphs of the time-varying impulse response in two ways. If `fl = 1`, `drawimp` outputs trajectories of impulse response for the horizons specified by `vt`. If `fl = 0`, `drawimp` outputs impulse response at the time points specified by `vt`. Note that `vt` should be a column vector whose size is less than or equal to four.

The TVP-VAR package provides two ways of computing responses, specified by the code `setvar('fastimp', fl)`. If `fl = 1` (*default*), time-varying impulse responses are computed using the posterior mean of time-varying parameters. This is a fast short-cut way of computing the responses. If `fl = 0`, the responses are computed every MCMC iteration and averaged at the end of iteration to calculate their posterior mean. The latter method significantly slows the computation, but it provides the estimates based on the joint posterior distribution of the responses and all parameters in the model.

The code `setvar('impulse', imax)` sets the maximum length (*default*:12) of impulse response: `imax` should be larger than or equal to all the values in `vt` of `drawimp(vt, 1)`. In the case of `setvar('fastimp', 0)`, a smaller `imax` yields faster computation.

## 2.3 Options and priors

The TVP-VAR package has the following options:

- `setvar('intercept', fl)`: excluding (*default*, `fl = 0`), or including (`fl = 1`) time-varying intercept;
- `setvar('fastimp', fl)`: sets fast computing of impulse responses. If `fl = 1` (*default*), time-varying impulse responses are computed using the posterior mean of time-varying parameters, while if `fl = 0`, the responses are computed every MCMC iteration and averaged as their posterior mean, which slows the computation;
- `setvar('impulse', imax)`: sets the maximum length (*default*:12) of impulse responses (if `fl = 0`, `imax` smaller, computation faster); note that `imax` should be larger than or equal to all the values in `vt` of `drawimp(vt, 1)`.
- `setvar('ranseed', j)`: the $j$-th ranseed is set.

The TVP-VAR package sets the default prior specification as

$$(\Sigma_\beta)_i^{-2} \sim \text{Gamma}(20, 10^{-4}), \quad (\Sigma_a)_i^{-2} \sim \text{Gamma}(4, 10^{-4}), \quad (\Sigma_h)_i^{-2} \sim \text{Gamma}(4, 10^{-4}),$$

where $IW$ denotes the invert Wishart distribution, $(\Sigma_a)_i$ and $(\Sigma_h)_i$ denote the $i$-th diagonal element of the matrices. In the TVP-VAR package, the hyperparameters in the prior distribution can be set by coding `setvar('prior',` *par*, *arg1*, *arg2*`)`. The first argument *par* is input as the string of either `'b'`, `'a'`, or `'h'`. If `setvar('SigB', 1)`, the default prior is $\Sigma_\beta \sim IW(25, 10^{-4}I)$; coding `setvar('prior')` corresponds to $IW($*arg1*, *arg2*$\times I)$.

## 2.4 Outputs

The TVP-VAR package outputs the following files in the procedure `MCMC(N)` and `DrawImp(·)`:

- `Figure(1)` – Results of MCMC draws (sample autocorrelations, sample paths, and posterior densities);
- `Figure(2)` – Posterior estimates of $\sigma_{it}^2 = \exp(h_{it})$ (posterior means and one-standard-deviation bands);
- `tvpvar_vol.xlsx` – Posterior estimates of $\sigma_{it}^2$ (posterior means and standard deviations);
- `Figure(3)` – Posterior estimates of $a_t$ (posterior means and one-standard-deviation bands);
- `tvpvar_a.xlsx` – Posterior estimates of $a_t$ (posterior means and standard deviations);

- `Figure(4)` – Posterior estimates of free elements in $A_t^{-1}$ ($\tilde{a}_t$; posterior means and one-standard-deviation bands);
- `tvpvar_ai.xlsx` – Posterior estimates of $\tilde{a}_t$ (posterior means and standard deviations);
- `Figure(5)` – (if `setvar('intercept',1)`) Posterior estimates of $c_t$ (posterior means and one-standard-deviation bands);
- `tvpvar_int.xlsx` – (if `setvar('intercept',1)`) Posterior estimates of $c_t$ (posterior means and standard deviations);
- `Figure(*)` – (if `drawimp(·)`) Posterior means of time-varying impulse response;
- `tvpvar_imp.xlsx` – Posterior means of time-varying impulse response. (Note: This file is output by `mcmc(N)` and used to draw impulse response by `drawimp`. Therefore, if the model is once estimated, you can re-draw impulse response by changing the horizons or time points without running MCMC again.)

# 3 Algorithm

See Nakajima (2011) for the details of the MCMC algorithm used in TVP-VAR package.

# References

Nakajima, J. (2011). Time-varying parameter VAR model with stochastic volatility: An overview of methodology and empirical applications. *Monetary and Economic Studies 29*, 107–142.
