# Supplementary material for: Source tracing and contagion measurement of carbon emission trading price fluctuation in China from the perspective of major emergencies
Source: PLoS One. 2024 Mar 8;19(3):e0298811. doi: 10.1371/journal.pone.0298811 (PMC10923469; doi:10.1371/journal.pone.0298811)
Supplement: S1 File — (ZIP) [file pone.0298811.s001.zip › supporting information files/wavelet/docs/_layouts/page.html]

---
layout: default
---

# {{ page.title }}

{{ content }}
