# Supplementary material for: Source tracing and contagion measurement of carbon emission trading price fluctuation in China from the perspective of major emergencies
Source: PLoS One. 2024 Mar 8;19(3):e0298811. doi: 10.1371/journal.pone.0298811 (PMC10923469; doi:10.1371/journal.pone.0298811)
Supplement: S1 File — (ZIP) [file pone.0298811.s001.zip › supporting information files/wavelet/docs/faq.html]

---
layout: default
title: Faq
category: faq
---

{% for post in site.categories[page.category] %}- {{ post.title }}
{% endfor %}
