# Supplementary figures and images for: Source tracing and contagion measurement of carbon emission trading price fluctuation in China from the perspective of major emergencies
Source: PLoS One. 2024 Mar 8;19(3):e0298811. doi: 10.1371/journal.pone.0298811 (PMC10923469; doi:10.1371/journal.pone.0298811)

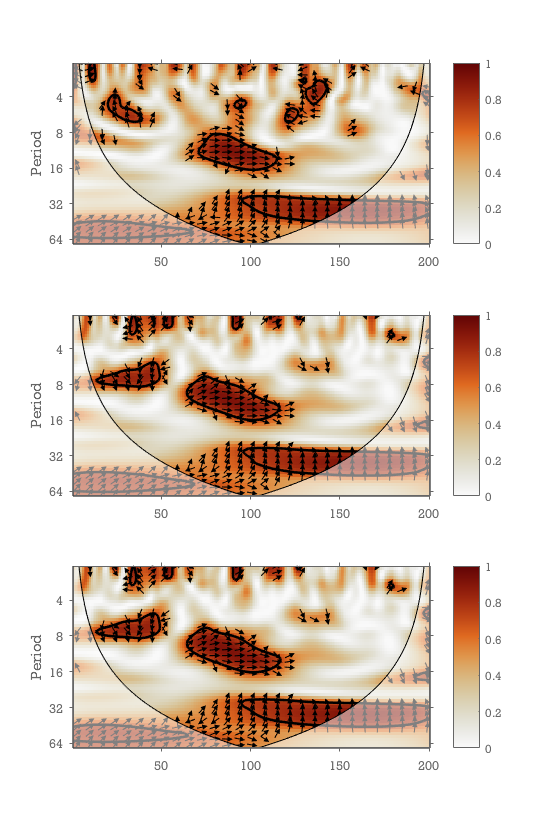

Supplement: S1 File — (ZIP) [file pone.0298811.s001.zip › supporting information files/wavelet/docs/images/ar1_in_wtc_01.png]

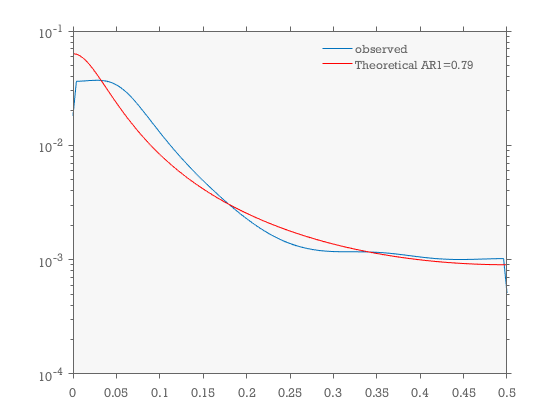

Supplement: S1 File — (ZIP) [file pone.0298811.s001.zip › supporting information files/wavelet/docs/images/is_ar1_ok_01.png]

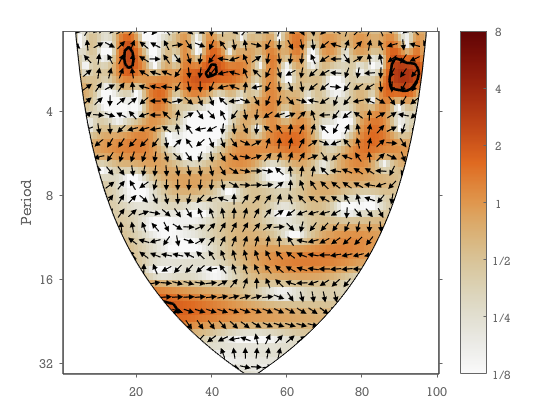

Supplement: S1 File — (ZIP) [file pone.0298811.s001.zip › supporting information files/wavelet/docs/images/matlab_renderer_bugs_01.png]

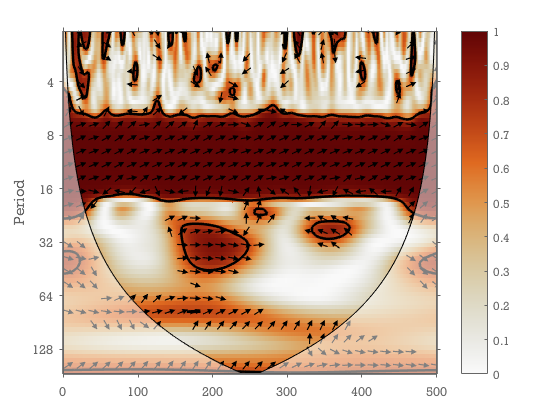

Supplement: S1 File — (ZIP) [file pone.0298811.s001.zip › supporting information files/wavelet/docs/images/no_monte_carlo_01.png]

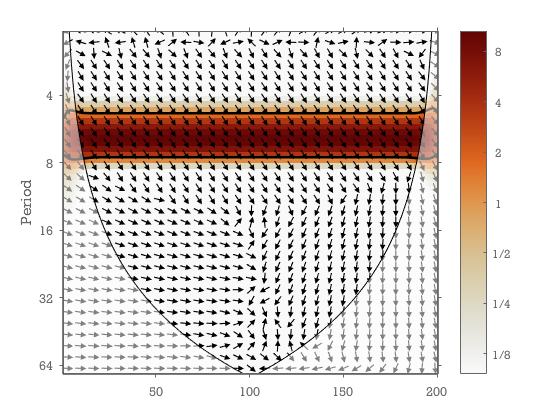

Supplement: S1 File — (ZIP) [file pone.0298811.s001.zip › supporting information files/wavelet/docs/images/phase_arrows_01.png]

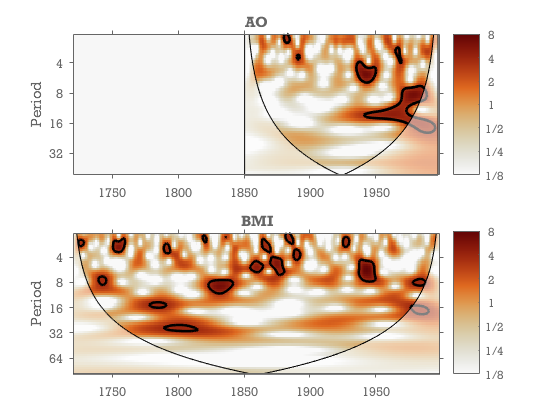

Supplement: S1 File — (ZIP) [file pone.0298811.s001.zip › supporting information files/wavelet/docs/images/wtcdemo_01.png]

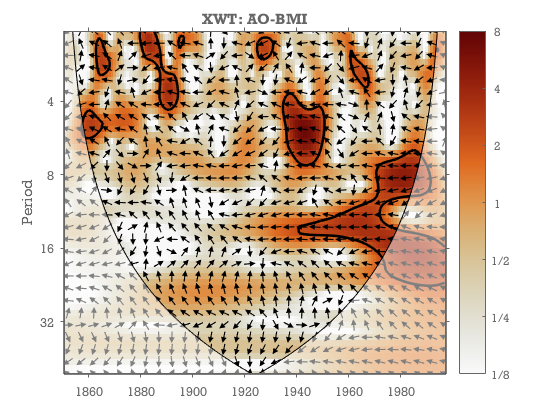

Supplement: S1 File — (ZIP) [file pone.0298811.s001.zip › supporting information files/wavelet/docs/images/wtcdemo_02.png]

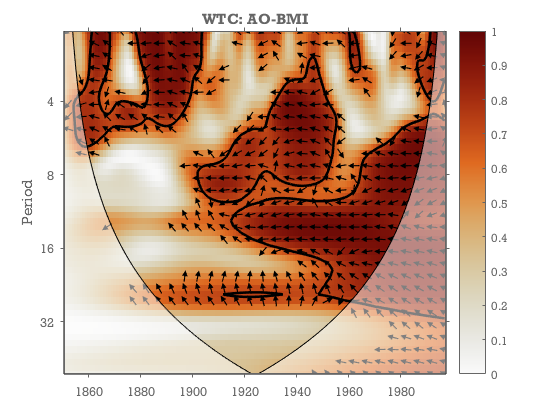

Supplement: S1 File — (ZIP) [file pone.0298811.s001.zip › supporting information files/wavelet/docs/images/wtcdemo_03.png]

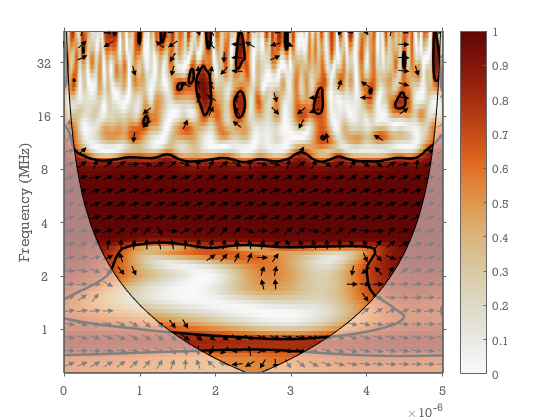

Supplement: S1 File — (ZIP) [file pone.0298811.s001.zip › supporting information files/wavelet/docs/images/yaxis_freq_vs_period_01.png]
